# Radiomics Features on Computed Tomography Combined With Clinical-Radiological Factors Predicting Progressive Hemorrhage of Cerebral Contusion

**DOI:** 10.3389/fneur.2022.839784

**Published:** 2022-06-14

**Authors:** Qingning Yang, Jun Sun, Yi Guo, Ping Zeng, Ke Jin, Chencui Huang, Jingxu Xu, Liran Hou, Chuanming Li, Junbang Feng

**Affiliations:** ^1^Department of Radiology, Chongqing University Central Hospital, Chongqing, China; ^2^Department of Research Collaboration, R&D Center, Beijing Deepwise & League of PHD Technology Co., Beijing, China; ^3^Department of Radiology, Panjiang Central Hospital, Guizhou, China

**Keywords:** traumatic brain injury, progressive hemorrhage, radiomics, machine learning, nomogram

## Abstract

**Background:**

Traumatic brain injury (TBI) is the main cause of death and severe disability in young adults worldwide. Progressive hemorrhage (PH) worsens the disease and can cause a poor neurological prognosis. Radiomics analysis has been used for hematoma expansion of hypertensive intracerebral hemorrhage. This study attempts to develop an optimal radiomics model based on non-contrast CT to predict PH by machine learning (ML) methods and compare its prediction performance with clinical-radiological models.

**Methods:**

We retrospectively analyzed 165 TBI patients, including 89 patients with PH and 76 patients without PH, whose data were randomized into a training set and a testing set at a ratio of 7:3. A total of 10 different machine learning methods were used to predict PH. Univariate and multivariable logistic regression analyses were implemented to screen clinical-radiological factors and to establish a clinical-radiological model. Then, a combined model combining clinical-radiological factors with the radiomics score was constructed. The area under the receiver operating characteristic curve (AUC), accuracy and F1 score, sensitivity, and specificity were used to evaluate the models.

**Results:**

Among the 10 various ML algorithms, the support vector machine (SVM) had the best prediction performance based on 12 radiomics features, including the AUC (training set: 0.918; testing set: 0.879) and accuracy (training set: 0.872; test set: 0.834). Among the clinical and radiological factors, the onset-to-baseline CT time, the scalp hematoma, and fibrinogen were associated with PH. The radiomics model's prediction performance was better than the clinical-radiological model, while the predictive nomogram combining the radiomics features with clinical-radiological characteristics performed best.

**Conclusions:**

The radiomics model outperformed the traditional clinical-radiological model in predicting PH. The nomogram model of the combined radiomics features and clinical-radiological factors is a helpful tool for PH.

## Introduction

Traumatic brain injury (TBI) is a disease with the highest mortality and disability rate in systemic trauma. It is the main cause of death and serious disability among the young adult population worldwide. The pathophysiological change in TBI is a dynamic process (1), and progressive hemorrhage (PH) is a significant cause of death and deterioration in TBI patients. It has been reported that the incidence rate of PH is approximately 18–64% (2–5). PH has an occult onset and a rapid progression, and when patients have the development of symptoms, the condition has already progressed to a certain extent, which brings difficulties for the clinical treatment. Moreover, PH directly affects the prognosis of patients, and prior cases (6, 7) have shown that the risk of developing a poor neurological outcome with PH was four times higher than that without PH. Therefore, early detection of PH is very important. At present, non-contrast CT is the first choice for brain trauma. It is very difficult to recognize PH by observing non-contrast CT images with the naked eye, and the clinical diagnosis of PH mainly depends on serial head CT scans. However, there are no unified guidelines on the CT reexamination time and frequency; blindly repeating head CT leads to unnecessary radiation exposure, and repeatedly moving the patients for examination can be harmful to them.

As a new technology, radiomics can extract many quantitative features from non-contrast CT images, can capture image information that cannot be evaluated by the naked eye, such as texture features and high-order features, and then can filter the quantitative features most relevant to the clinical findings by statistical methods. Radiomics has been widely used for evaluation of the central nervous system, such as tumors (8–11), the prediction of hematoma enlargement in hypertensive intracerebral hemorrhage (12, 13), the classification of intracranial aneurysm rupture (14), and the etiological classification of intracranial hematoma (15, 16). However, few studies have reported the application of radiomics in progressive hemorrhage of cerebral contusion.

The purpose of this retrospective study was to seek the optimal radiomics model based on non-contrast CT scanning to predict PH by applying various machine learning methods and comparing the prediction performance of this model with the traditional model based on clinical factors and radiological factors.

## Materials and Methods

### Study Population

This study was approved by the medical ethics committee of the Chongqing University Central Hospital, and the requirements for informed consent were waived.

**Figure 1:**
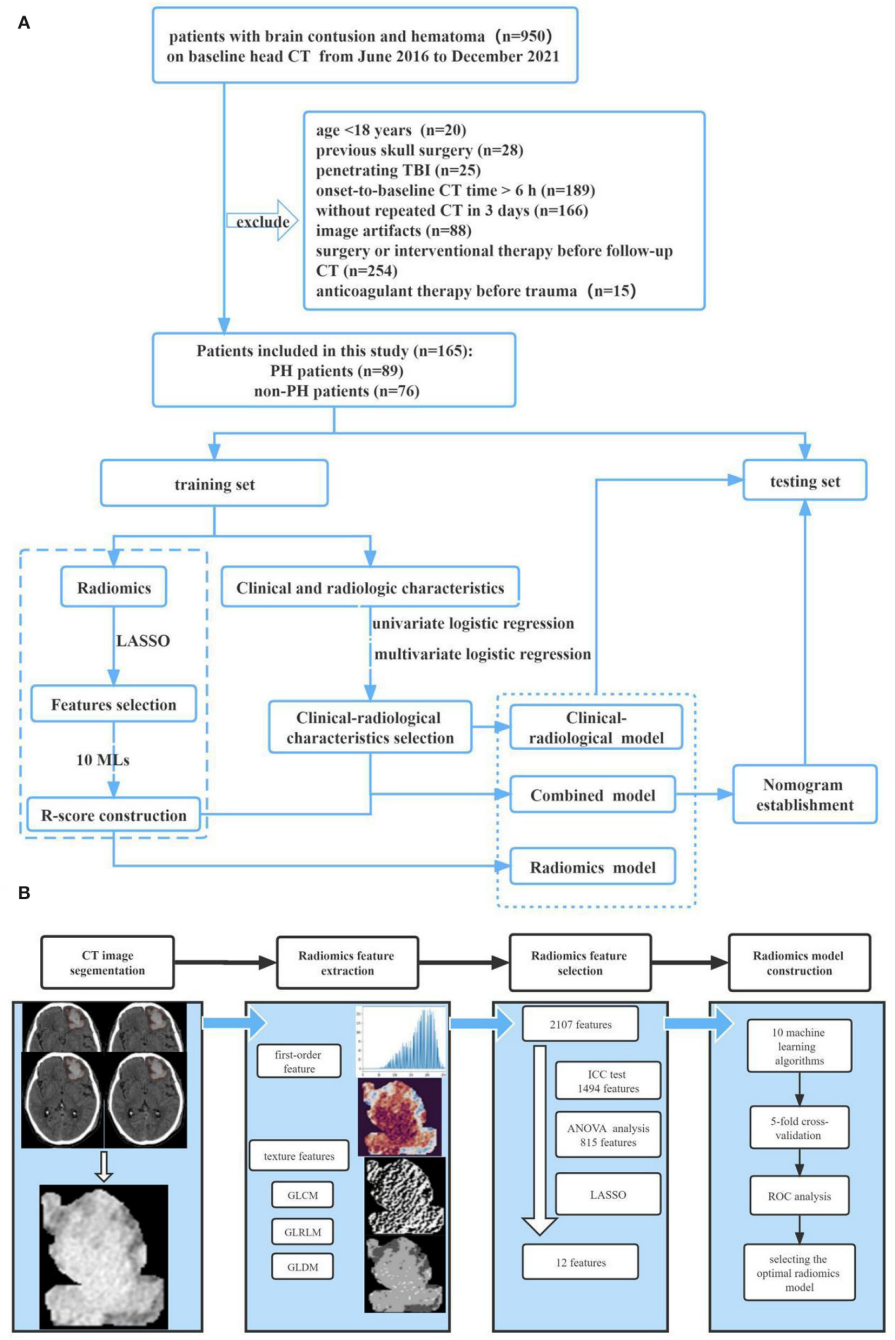
Study overview. **(A)** Workflow of the study. **(B)** Workflow of the radiomics analysis.

Patients with a brain contusion and hematoma on baseline head CT after trauma were recruited from June 2016 to December 2021 at the Chongqing University Central Hospital. The exclusion criteria were as follows: (1) patients <18 years old; (2) patients with a history of skull surgery; (3) patients with a baseline head CT more than 6 h after trauma; (4) patients who did not undergo repeated CT within 3 days of baseline; (5) severe artifacts on the baseline images; (6) patients with penetrating TBI; (7) patients with brain surgery or interventional therapy before the follow-up head CT; and (8) patients with anticoagulant therapy before trauma; and (9) patients with non-traumatic brain diseases, such as tumor, discovered on CT. The patient screening process is shown in Figure 1. After the exclusion criteria screening was applied, 165 cases with TBI were selected, including 89 cases with PH and 76 cases without PH. After admission, all patients were evaluated and treated according to the Clinical Guidelines for the Management of Head Injury (17).

The clinical-radiological data (age, sex, Glasgow Coma Scale score) were acquired from Picture Archiving and Communication Systems and the patients' medical records. PH was defined as the increase of the volume of intracranial hematoma by 25% or more when compared with the initial CT (18). The hematoma volume was calculated as ABC/2.

The head CT images were from United Imaging 760 64 Shanghai China and General Electric Company, LightSpeed 64, America. All the CT scanning parameters were similar: a tube voltage of 120 kV, a tube current of 200 mA, an axial technical section thickness of 5 mm, and a reconstruction interval of 1.25 mm.

To ensure that the labeling ratio was not affected, the data were randomly divided into training and testing sets at 7:3. Since some patients had multiple independent lesions, namely 30 patients (18.29%) had two lesions, 5 patients (3.05%) had three lesions, and 1 patient (0.61%) had four lesions, samples from the same patient were assigned to the same cohort to avoid data leakage. Finally, we randomly assigned lesions into a training set (*n* = 145) and a testing set (*n* = 62). Models were fitted on the training set and were independently tested on the testing set.

### Image Segmentation and Feature Extraction

All regions of interest (ROIs) were delineated around the boundary using a postprocessing platform (Dr. Wise Multimodal Research Platform1 v1.6.3, Beijing Deepwise & League of PHD Technology Co., Ltd, Beijing, China). An open-source package of Python, called “PyRadiomics” (19), was used to generate the radiomics data from the original images. The software package could extract signal strength features, texture features, shape features, and higher-order features from the original images. Higher-order features are calculated from the above features using filters (wavelet, Laplace-Gauss, square root, logarithm, exponent, gradient transform, and local binary mode transform). All the calculation formulas and the pipeline can be found at https://pyradiomics.readthedocs.io/en/latest/. In total, 2,107 radiomics features were extracted automatically, including 414 first-order features, 14 shape features, 506 gray level co-occurrence matrix (GLCM) features, 368 gray level size zone matrix (GLSZM) features, 368 gray level run length matrix (GLRLM) features, 322 gray level dependence matrix (GLDM) features, and 115 neighboring gray-tone difference matrix (NGTDM) features.

The values of each training set feature were standardized with the Z score, and a similar normalization procedure, which utilized the mean and standard deviation values of the training set, was applied to the testing set.

### Radiomics Feature Selection

First, we applied intraclass coefficient (ICC) analysis to identify features with high reproducibility and consistency of measurements. Reader 1 and Reader 2 independently redelineated the original images, and for each reader, 17 patients were randomly selected. Features with an ICC > 0.75 were selected for further analysis. After that, an analysis of variance (ANOVA) model was built, and it excluded features without significant differences between the PH group and the control group. Finally, we used the least absolute shrinkage and selection operator (LASSO) method, whose penalty parameter was tuned by a 5-fold cross-validation (CV).

The whole variable reduction process above was performed on the training set.

### Machine Learning and Radiomics Signature Construction

We implemented 10 different machine learning (ML) algorithms—logistic regression (LR), support vector machine (SVM), K-nearest neighbors (KNN), linear discriminant analysis (LDA), quadratic discriminant analysis (QDA), Gaussian naive Bayes (GNB), artificial neural network (ANN), random forest (RF), XGBoost, and CatBoost—on the training dataset and determined the optimal hyperparameters of these models by the 5-fold CV method and grid search technique. For each selected model, receiver operating characteristic (ROC) curves were generated, and the average area under the curve (AUC) was calculated to assess the prediction performance. We used a learning curve to determine the overfitting of the model. A model was considered overfitted if its AUC for the training set was significantly higher than that for the validation set. Finally, with the best ML classifier, we built a radiomics model on the whole training set and constructed a radiomics signature (Rad-score) using the logit function


$$\begin{array}{l} \text{rad}-\text{score}=\text{ln}\frac{\text{p}}{1-\text{p}} \end{array}$$

where, p refers to the probability of PH predicted by our radiomics model for each patient. We also calculated the accuracy, F1 score, sensitivity, and specificity for comparison.

### Clinical-Radiological Analysis

To select the clinical-radiological characteristics with significant differences between the PH group and control group, we performed the chi-square test for categorical variables and the Mann–Whitney U-test or Student's *t*-test for continuous variables. Then, univariate logistic regression and multivariable logistic regression were implemented to remove those features without significance and to determine the final clinical-radiological factors that were used to establish the clinical (LR) model.

### Combined Model Establishment

A combination of the radiomics signature from the radiomics model and clinical-radiological signatures from the clinical-radiological model was used to conduct the combined model by the LR algorithm to provide a nomogram to evaluate the risk of PH. The ROC-AUC, accuracy, F1-score, sensitivity, and specificity metrics were calculated separately on the training set. The testing data was used to assess the performance of these three classifiers, and the decision curve and the calibration curve were also illustrated for comparison. A Youden index analysis was constructed to determine the optimal threshold (cutoff value) of the critical probability.

### Statistical Analysis

“Scipy.stats,” an open-source package of Python, was used for all statistical tests (20). Categorical variables were compared by the chi-square test. The Shapiro–Wilk's test was performed to assess the normality of continuous variables. For variables with the null hypothesis rejected, we used the Mann–Whitney U-test to test for feature significance. For variables whose *p*-values were higher than 0.05, these variables followed a normal distribution, and then we performed the Levene test. If the results were significant, Welch's test was conducted. If the results were not significant, it was considered that the variances were equal, and Student's *t*-test was utilized. Differences in the AUC values between the different classifiers were compared using DeLong's test. A two-sided *p*-value < 0.05 was considered statistically significant. Hosmer–Lemeshow tests were used to calculate if the observed event rates matched the expected event rates in the population subgroups.

## Results

### Patients' Characteristics

The clinical-radiological factors of the PH group and non-PH group are given in Table 1. The proportion of the PH group in the training set was 51.03% (74 of 145), and that in the validation set was 53.23% (33 of 62). In the training set, the factors including scalp hematoma, the GCS score, the onset-to-initial CT time, and fibrinogen level were significantly different between the two groups (*p* < 0.01). The other features were not significant (*p* > 0.05).

**Table 1:** Comparisons of patient characteristics in the training set and testing set.

	**Training set (*****n*** **=** **145)**			**Testing set (*****n*** **=** **62)**		
	**PH (*n* = 74)**	**Non-PH (*n* = 71)**	***p value***	**PH (*n* = 33)**	**Non-PH (*n* = 29)**	***p value***
Sex, male	14 (18.9)	12 (16.9)	0.999	13 (39.4)	3 (10.3)	0.147 ^b^
Multiple primary brain contusions	52 (70.3)	49 (69.0)	1.000	28 (84.8)	25 (86.2)	1.000 ^b^
Craniofacial fracture	67 (90.5)	59 (83.1)	0.779	33 (100.0)	28 (96.6)	0.885 ^b^
Epidural hematoma	16 (21.6)	12 (16.9)	0.972	12 (36.4)	4 (13.8)	0.392 ^b^
Subdural hematoma	67 (90.5)	54 (76.1)	0.239	28 (84.8)	25 (86.2)	1.000 ^b^
Subarachnoid hemorrhage	67 (90.5)	58 (81.7)	0.665	33 (100.0)	27 (93.1)	0.671 ^b^
Midline shift	3 (4.1)	0 (0.0)	0.568	3 (9.1)	0 (0.0)	0.597 ^b^
Scalp hematoma	73 (98.6)	58 (81.7)	0.018*	33 (100.0)	26 (89.7)	0.465 ^b^
Diabetes^a^	6 (8.1)	10 (14.1)	0.858	6 (18.2)	8 (27.6)	0.941 ^b^
Hypertension^a^	22 (29.7)	19 (26.8)	0.997	14 (42.4)	5 (17.2)	0.330 ^b^
Smoking	16 (21.6)	22 (31.0)	0.801	6 (18.2)	8 (27.6)	0.941 ^b^
Alcoholism	19 (25.7)	24 (33.8)	0.887	7 (21.2)	15 (51.7)	0.179 ^b^
Age, mean ± SD (years)	56.5 ± 17.742	59.155 ± 18.245	0.305	59.848 ± 18.346	57.862 ± 16.487	0.657 ^c^
INR^a^, mean ± SD	1.076 ± 0.187	1.061 ± 0.148	0.811	1.09 ± 0.116	1.1 ± 0.134	0.799
Admission SBP^a^, mean ± SD (mmHg)	142.446 ± 21.144	148.408 ± 27.622	0.146 ^c^	144.727 ± 28.627	138.69 ± 30.844	0.427 ^c^
GCS score^a^, mean ± SD	12.365 ± 2.356	12.958 ± 2.381	0.011*	11.242 ± 2.948	11.931 ± 3.023	0.072
Platelet, mean ± SD (109/L)	170.892 ± 57.15	180.535 ± 53.678	0.097	161.152 ± 42.854	176.897 ± 49.703	0.185 ^c^
APTT^a^, mean ± SD (s)	33.705 ± 4.486	33.615 ± 4.018	0.970	32.391 ± 2.567	35.379 ± 4.631	0.004 ^d^
Prealbumin, mean ± SD (mg/L)	241.689 ± 59.41	239.38 ± 56.184	1.000	237.394 ± 67.875	232.931 ± 61.04	0.832
Onset-to-CT time^a^, mean ± SD (h)	2.313 ± 1.345	2.984 ± 1.764	0.029*	2.326 ± 1.219	3.441 ± 1.921	0.062
First-to-second CT time, mean±SD (h)	22.53 ± 20.38	21.66 ± 18.57	0.182	20.11 ± 18.13	20.11 ± 20.79	0.554
Admission DBP^a^, mean ± SD (mmHg)	84.541 ± 12.456	86.704 ± 14.395	0.147	84.485 ± 17.566	83.759 ± 13.543	0.761
Fibrinogen, mean ± SD (g/L)	2.483 ± 0.788	5.708 ± 13.488	0.003*	2.419 ± 0.623	2.897 ± 0.993	0.025 ^c^

^a^*Diabetes, Diabetes History; Hypertension, Hypertension history; INR, International normalized ratio; Admission SBP, Systolic blood pressure on admission; GCS score, Glasgow coma scale score; APTT, Activated partial thrombin time; Onset-to-CT time, Interval from trauma to first CT; Admission DBP, Diastolic blood pressure on admission*.

^b^ For categorical factors, the chi-squared test was used, data in parentheses are percentages. ^c^Student's t-test. ^d^ Welch's t-test. For other continuous factors, the Mann–Whitney U-test was used, and for categorical factors, the chi-squared test was used. * Predictors included for further analysis (p < 0.05).

### Radiomics Feature Selection

**Figure 2:**
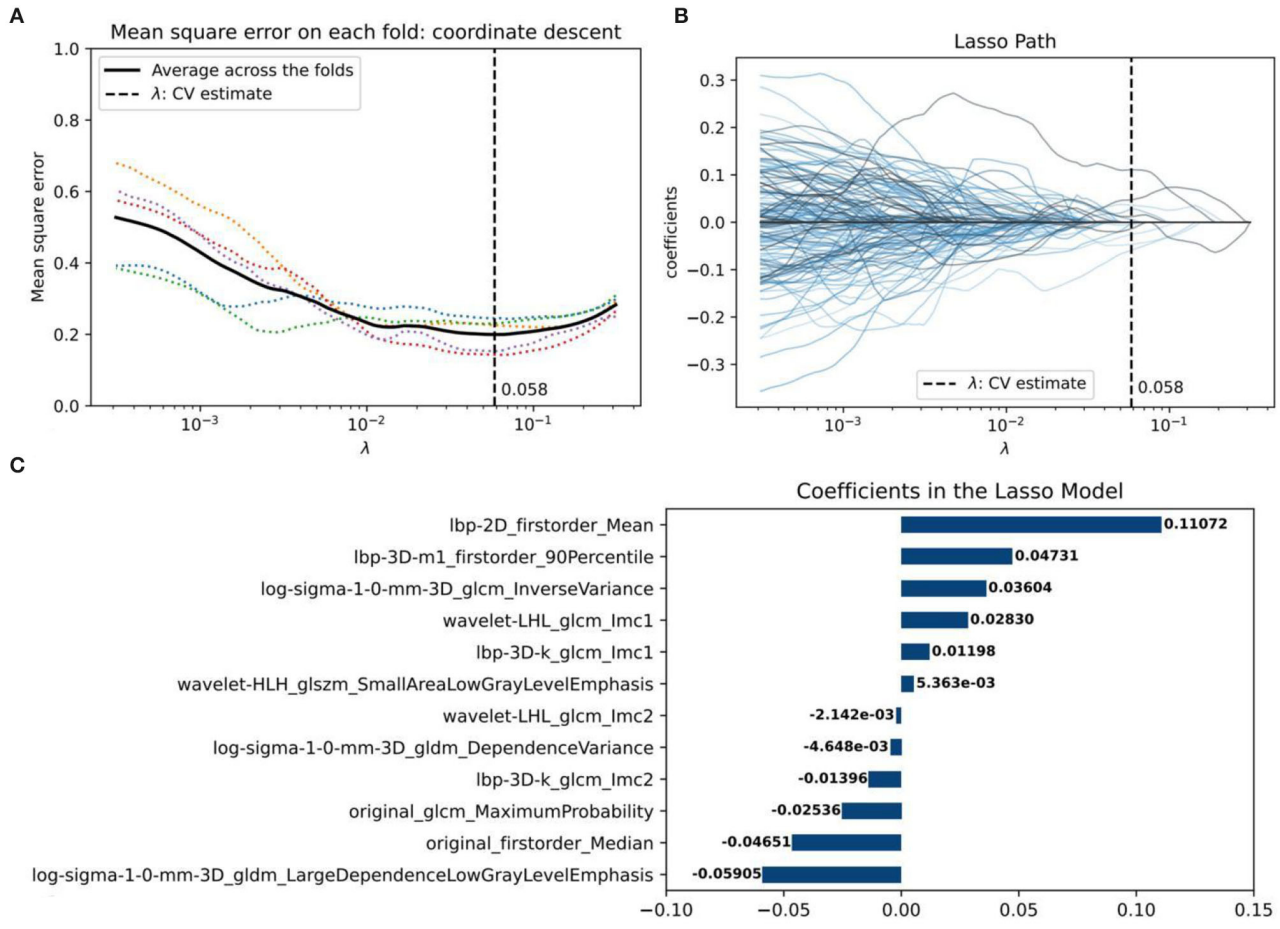
The radiomics feature selection using the least absolute shrinkage and selection operator (LASSO) regression algorithm. **(A)** The coefficient lambda of the penalty term in LASSO was seen as a hyperparameter and was tuned *via* the 5-fold cross-validation (CV) method. The x-axis represents the values of lambda. The black curve represents the average mean square error (MSE) for each model given lambda. The vertical line marks the value of the best lambda, which is 0.058 because, at this time, the average MSE reaches its lowest point. **(B)** Radiomics feature coefficient reduction path curves. Finally, 12 non-zero factors were selected. **(C)** The histogram of the resulting radiomics feature coefficients from the optimal LASSO model.

After intra- and interreader ICC analysis, a total of 1,494 radiomics features were considered robust and consistent. After that, 815 factors showed significant differences between the PH group and control groups (*p* < 0.05) *via* ANOVA. These features were finally reduced to 12 through the LASSO model (Figure 2).

### Machine Learning and Radiomics Signature Construction

**Figure 3:**
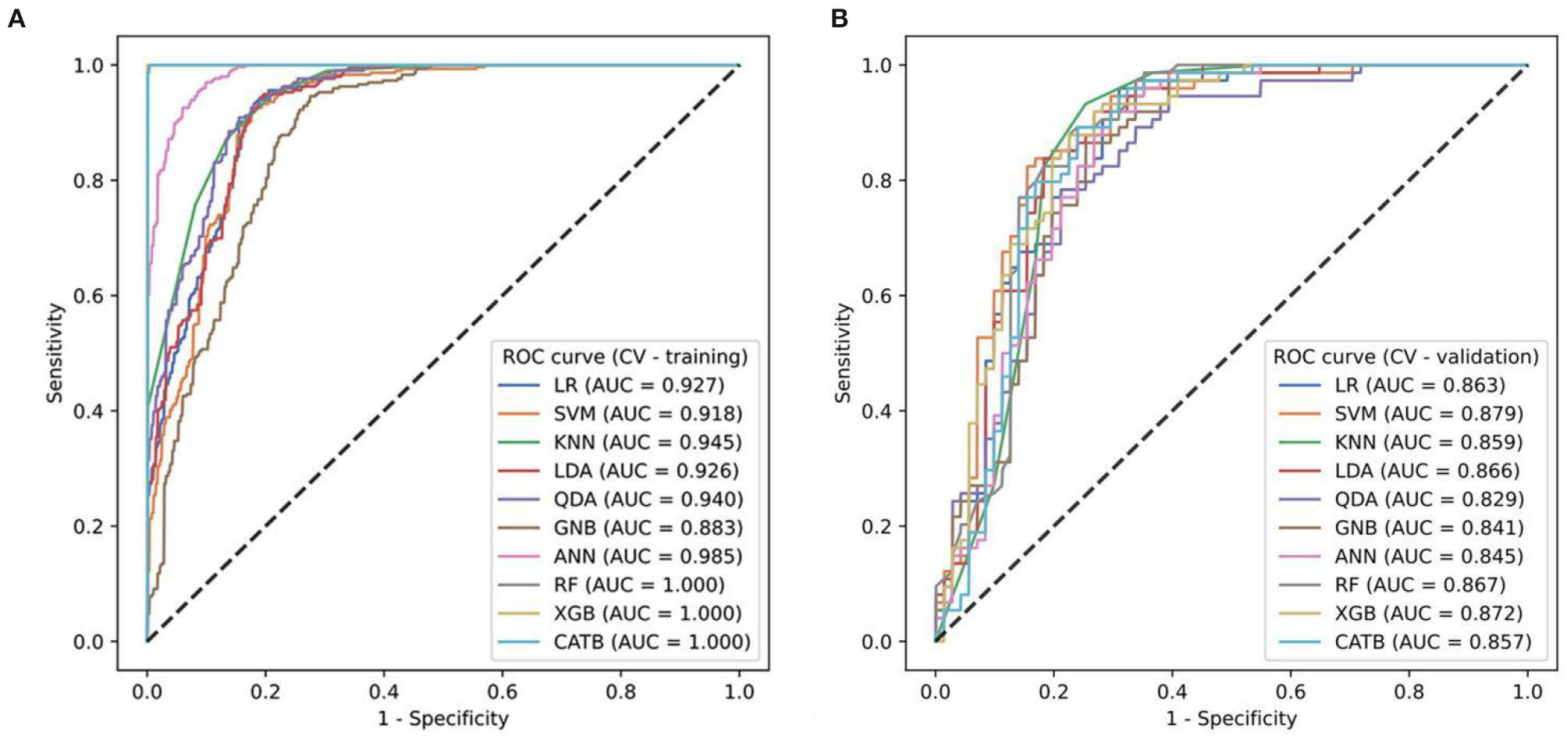
**(A,B)** The ROC curves of ten ML models for predicting progressive hemorrhage of cerebral contusion.

To find the optimal radiomics model to establish the Rad-score, we implemented ten machine-learning algorithms on the training set and compared their predictive ability by calculating commonly used metrics, including the ROC-AUC, accuracy, F1-score, sensitivity, and specificity (Figure 3). Using the 5-fold cross-validation (CV) method, we evaluated the performance of several classifiers in the training phase (CV training) and validation phase (CV validation). In addition to GNB, the other models performed well on the CV training. However, the two tree models of RF and Xgboost overfitted the CV training. The SVM model had the best predictive ability on the CV validation (AUC = 0.918), and there was no overfitting compared to the result on the CV training (AUC = 0.879). Therefore, the SVM algorithm was determined as our radiomics model and was used to generate the radiomics scores (Rad-score) on the training set and testing set.

### Clinical-Radiological Model

The results of the univariate and multivariable LR are listed in Table 2. After that, scalp hematoma, the onset-to-CT time, and fibrinogen level, whose coefficients were significant in both univariate LR (*p* = 0.008, *p* = 0.013, and *p* = 0.014, respectively) and multivariable LR (*p* < 0.0001, *p* = 0.007, and *p* = 0.010, respectively), were determined as the variables to be used in the clinical-radiological model.

**Table 2:** Risk factors for progressive hemorrhage.

	**Univariate logistic regression** ^**b**^		**Multivariable logistic regression**	
	**Odds ratio**	***p value***	**Odds ratio**	***p value***
Scalp hematoma	16.362 (2.079, 128.757)	0.008	10.382 (3.595, 29.978)	<0.0001*
GCS score, mean ± SD	1.0 (0.975, 1.025)	0.974	/	/
Onset-to-CT time^a^, mean ± SD (h)	0.759 (0.611, 0.943)	0.013	0.728 (0.578, 0.915)	0.007*
Fibrinogen, mean ± SD (g/L)	0.607 (0.407, 0.905)	0.014	0.638 (0.453, 0.898)	0.010*

^a^*Onset-to-CT time, Interval from trauma to first CT*.

^b^*Univariate LRs of the clinical factors on the labels were implemented one by one to check whether there were insignificant variables*.

*The predictors included for the further analysis (p < 0.05).

### Combined Model Establishment

**Figure 4:**
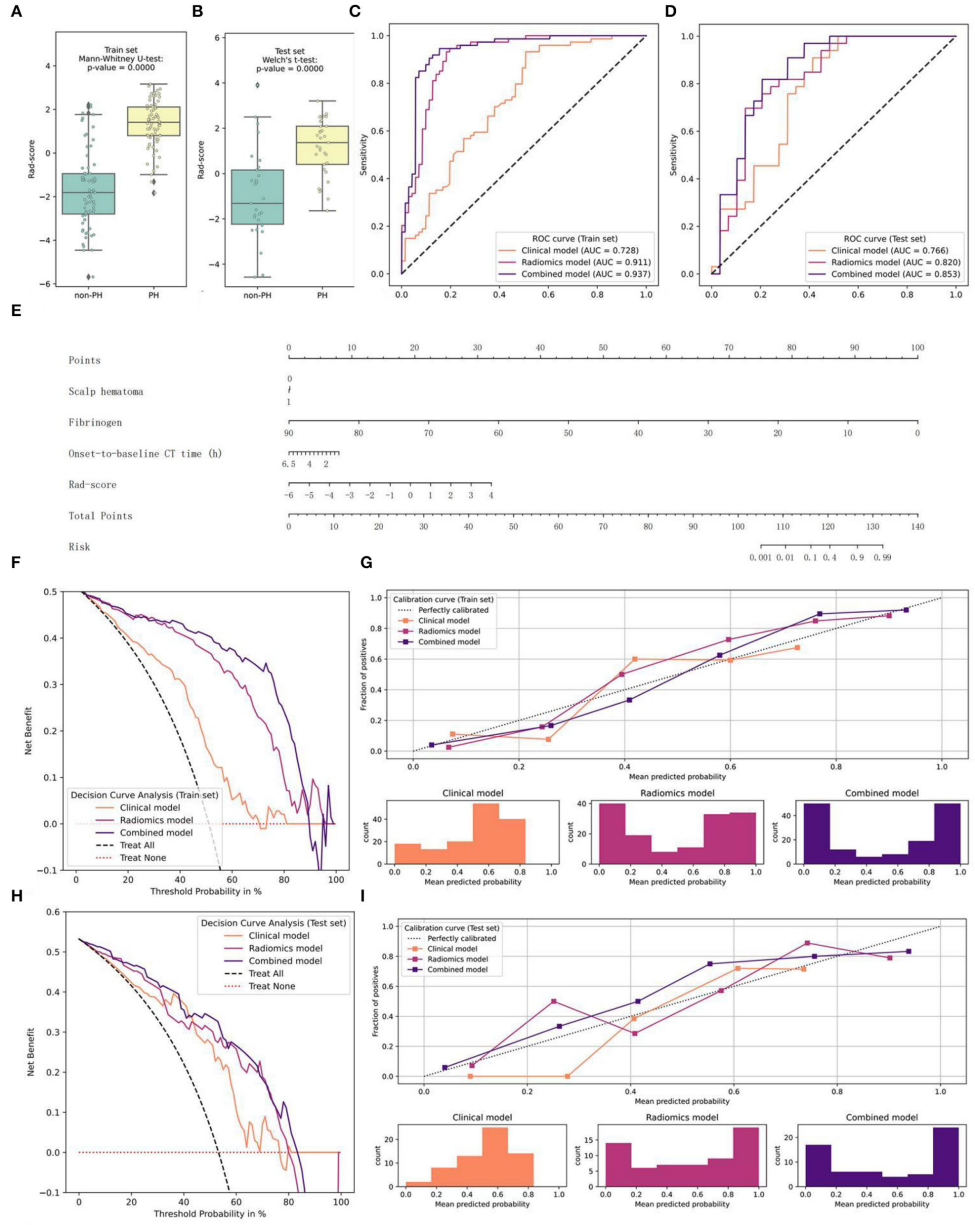
Graphs of the construction of the combined model, the nomogram, and the model comparison. **(A,B)** The distribution of the Rad-score and the Mann–Whitney U-test results between the two groups on the training and testing sets **(E)** Nomogram **(C–I)** Performance of the clinical-radiological, radiomics, and combined models on the training and testing sets **(C,D)** ROC curves and AUC values **(G,I)** Calibration curves **(F,H)** Decision curves.

We then added the Rad-score to the clinical-radiological model; that is, radiomics and clinical-radiological signatures were used to develop a combined signature. On the training set, we implemented an LR algorithm and built a nomogram as the combined classifier, made predictions on the testing set, and assessed the performance of the clinical-radiological, radiomics, and combined models by calculating metrics including the ROC-AUC, accuracy, F1-score, sensitivity, and specificity (Figure 4; Table 3).

**Table 3:** Comparisons of predictive models on the training and testing set.

**Model**	**vs. the Combined model^**b**^**	**AUC (95% CI)**	**Accuracy**	**F1-score**	**Sensitivity**	**Specificity**	**Threshold^a^**
**Training set**							
Clinical model	<0.00001****	0.728 (0.660–0.799)	0.717	0.771	0.905	0.493	0.427
Radiomics model	0.0618	0.911 (0.865–0.951)	0.876	0.885	0.905	0.817	0.376
Combined model	/	0.937 (0.897–0.970)	0.897	0.899	0.932	0.887	0.545
**Testing set**							
Clinical model	0.0179 *	0.766 (0.663–0.871)	0.726	0.779	0.909	0.517	/
Radiomics model	0.0456*	0.820 (0.724–0.906)	0.758	0.805	0.939	0.552	/
Combined model	/	0.853 (0.765–0.930)	0.806	0.818	0.818	0.793	/

^a^*On the training set, a Youden index analysis was constructed to determine the optimal threshold (cut-off value) of the classification probability and was used to predict the outcomes based on the testing set*.

^b^p-values of DeLong's test; * p < 0.05; **** p < 0.0001.

The combined model fit best on the training set (AUC = 0.937), and the results of DeLong's test showed significant differences between this and the other two models. In addition, the radiomics model was significantly better than the clinical-radiological model (*p* = 0.0002). In the other metrics, the performance of the combined model was the best. On the testing set, the score attained by the combined model (AUC = 0.853) was still significantly higher than that of the other two classifiers. Except for the specificity, the other indicators of the nomogram all ranked first. From the perspective of the calibration curve, the combined model was closer to the ideal on both the training and testing sets; from the view of the decision curve, using the nomogram to predict the PH would be more beneficial than that without the Rad-score or the Radiomics model. Furthermore, only the combined model showed good results (*p* = 0.4208>0.2) when the HL test was conducted in the training set, and the *p*-values of the clinical-radiological and radiomics models were both < 0.05. In the testing set, the HL statistics indicated a poor fit for the clinical-radiological model (*p* = 0.0158 < 0.05). The radiomics and the combined models adequately fitted the data (*p* = 0.1686 and *p* = 0.1834 > 0.05).

## Discussion

In this study, we used 10 different MLs to construct the radiomics models and to select the best model. We established the other two models to predict PH, including the traditional clinical-radiological model and the combined model (integrating clinical-radiological factors with radiomics features) and we compared the prediction performance of the three models. Our results suggest that the radiomics model is better than the clinical-radiological model, and the combined model is the best of all models. Finally, we established a convenient prediction nomogram based on the combined model to assist neurosurgeons and radiologists in identifying PH.

Many clinical and radiological factors have been demonstrated to be predictors of PH. Some studies (2, 3, 18, 21–24) have suggested that hypertension, the triglyceride level, D-dimer level, fibrin monomers, the onset-to-baseline CT time, the initial volume of contusion, the coexistence of SAH or SDH on baseline CT, skull fracture, and the “spot sign” formed by contrast extravasation on baseline CTA were independent predictors of PH. In this study, the first result we found was that fibrinogen was an independent predictor of PH, which might be attributed to the hyperfibrinolysis that is induced by a TBI. In the early stage of TBI, the blood is in a hypercoagulable state due to the activation of the coagulation pathway. However, the activation of the protein C system, the consumption of platelets, the decline of platelet function, the depletion of coagulation factors, and the activation of endothelial cells lead to hyperfibrinolysis, and fibrinogen decreases over time. Previous studies have confirmed that fibrinolysis is related to the progression of bleeding in TBI patients. As a retrospective study, the clinical data of some patients were incomplete (D-dimer, blood lipid, etc.). Therefore, the impact of these clinical data on PH could not be assessed, which is a limitation of this study. The second factor in the clinical-radiological model was time. The shorter the onset-to-baseline CT time was, the higher the incidence of PH, and it was an independent predictor of PH, which was consistent with previous studies (25). In addition, we also found that scalp hematoma was another independent predictor of PH. Some patients without a skull fracture but who had a scalp hematoma developed PH in our study. Scalp hematoma was also a manifestation of the severity of trauma and was a radiological factor that cannot be ignored. We did not find any literature report that scalp hematoma was an independent predictor of PH, which might be because the previous relevant literature (2, 3, 5, 7, 26) did not include scalp hematoma in its studies. In the past, researchers had established some models to predict PH based on clinical and radiological factors, but the accuracy of these models was relatively low, ranging from 0.69 to 0.77 (2, 18, 27). Like our clinical-radiological model, a low accuracy meant that patients at risk of PH could not be accurately selected.

Radiomics analysis has great potential in hemorrhagic diseases and is helpful for the etiological diagnosis of intracerebral hemorrhage and for detecting hematoma expansion in cases of spontaneous intracerebral hemorrhage (28). However, it is unclear whether radiomics features can predict the development of traumatic PH. Our study indicated the value of radiomics signatures in detecting PH. We screened 12 out of 2,107 extracted radiomics features for modeling, including 3 first-order features, 6 GLCM features, 1 GLSZM feature, and 2 GLDM features. Among these radiomics features, the first-order features reflected the heterogeneity of the hematoma density by describing the voxel intensity distribution and internal variation degree. The GLCM, GLSZM, and GLDM features reflected the heterogeneity of the hematoma density by describing the irregular texture features of the hematoma. The change in the hematoma density depended on the time course of bleeding. When progressive hemorrhage occurs and when there is a greater change in the hematoma density, there is a greater heterogeneity of the hematoma density. Therefore, brain trauma with PH has greater heterogeneity than stable brain trauma. A previous study (29) has suggested that the attenuation of a heterogeneous hematoma is a predictor of hematoma expansion and might reflect active bleeding. Then, we applied 10 ML algorithms to establish the omics model. Compared with the traditional models such as LR, the ML algorithms are better at processing multidimensional features, because, from the data, they can identify some potential patterns that in most scenarios are not linear or polynomial, so LR is not always the most reasonable choice. Our study supports these data (Figure 3). The SVM classifier we finally chose used a sigmoid kernel function to map our data to higher dimensions and then distinguished it with a hyperplane that allows the model to learn richer non-linear patterns of the relationship between the PH group and the non-PH group. Thus, our results revealed that the radiomics model had a better discrimination ability than the traditional clinical-radiological model.

**Figure 5:**
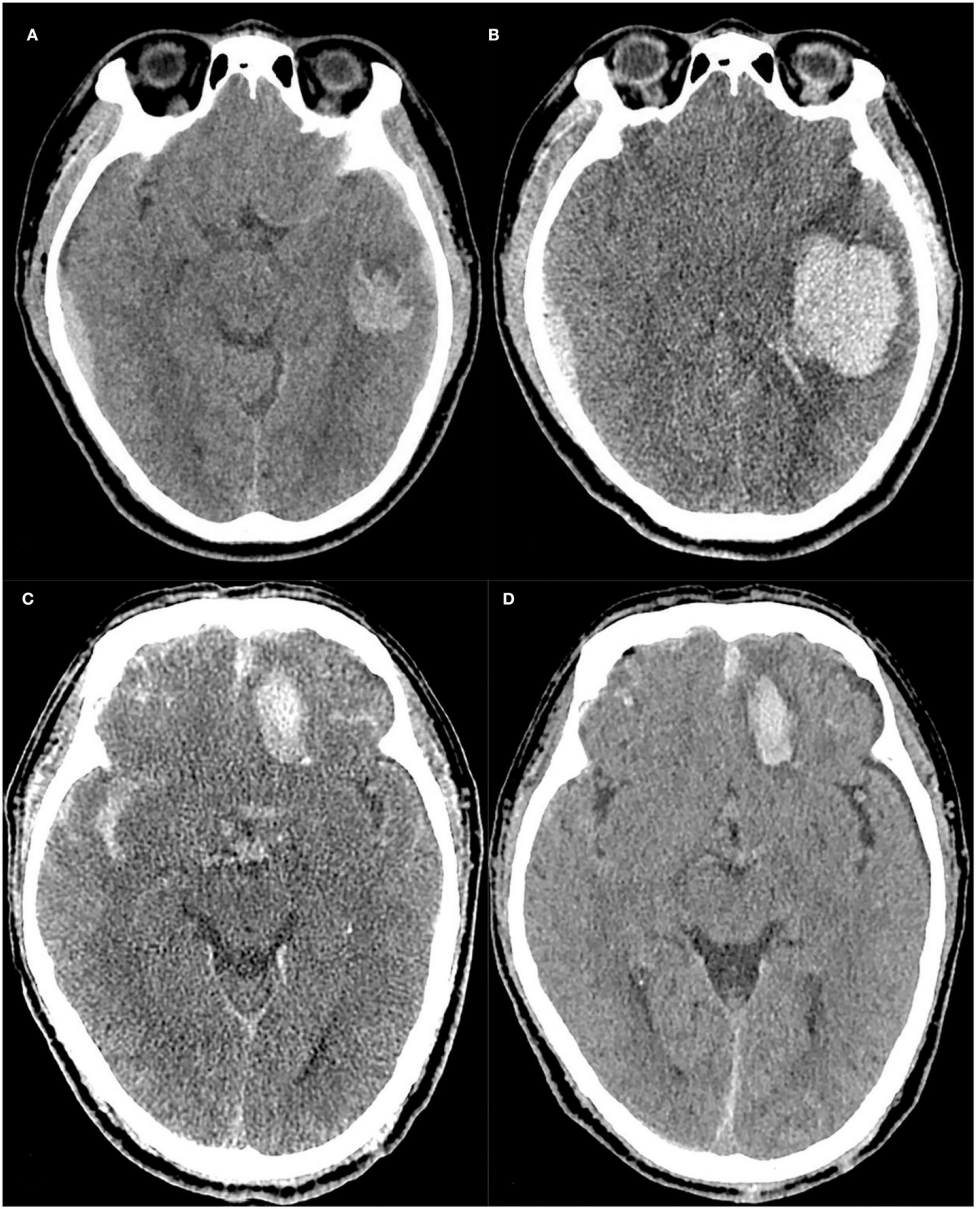
Case 1: A case with progressive hemorrhage, The baseline and follow-up CT images of a TBI patient **(A,B)**, Scalp hematoma = 1, Fibrinogen = 2.68, Onset-to-CT time = 2 h, R-score = 1.925. The risk of PH calculated by the nomogram was approximately 94%, and the hematoma volume was expanded to approximately 42.9 ml on the follow-up 10-h CT compared to 10.5 ml on the baseline CT. Case 2: A case without progressive hemorrhage, The baseline and follow-up CT images of another TBI patient **(C,D)**, Scalp hematoma = 1, Fibrinogen = 3.01, Onset-to-CT time = 6 h, R-score =-1.144. The risk of PH calculated by the nomogram was approximately 0.044%. At 17 h after trauma, the hematoma volume had not expanded.

To find the best predictive PH model, we constructed a combined model integrating the Rad-score with clinical-radiological factors. As we expected, compared with the clinical-radiological and radiomics models, the combined model showed the best discrimination and sensitivity in detecting PH. The nomogram was a more objective, convenient, and rapid personalized tool to predict PH based on the combined model (Figure 5). It helps neurosurgeons to identify PH patients and to revise treatment schemes in time, reduces mortality, and improves the prognosis of patients. At the same time, it solves the problem of blind repeat CT and reduces the waste of medical resources and unnecessary radiation exposure. At present, our research has some limitations. First, it is a single-center retrospective study, and it lacks external validation of data from other centers. Second, because some patients had multiple lesions, we assigned data belonging to the same patient to the same set to avoid data leakage, but the fly in the ointment is that these samples may cause a local autocorrelation in the model. Finally, when a hematoma is in the cerebral cortex, there may be an inaccurate separation on CT due to the partial solvent effect.

## Conclusions

In this study, the radiomics model outperformed the traditional clinical-radiological model in predicting PH. A nomogram model of combined radiomics features and clinical-radiological factors could offer an efficient and convenient tool for PH.

## Data Availability Statement

The raw data supporting the conclusions of this article will be made available by the authors, without undue reservation.

## Ethics Statement

The studies involving human participants were reviewed and approved by the Medical Ethics Committee of Chongqing University Central Hospital. Written informed consent from the patients/participants or patients/participants' legal guardian/next of kin was not required to participate in this study in accordance with the national legislation and the institutional requirements.

## Author Contributions

YG, QY, PZ, and JS designed and managed. QY, JS, LH, and JF collected the clinical and CT data. KJ, CH, and JX analyzed all data and technical support. YG and QY drafted the manuscript. CL, PZ, and JS revised the manuscript. YG, QY, PZ, JS, and CL approved for the final manuscript. All authors contributed to the article and approved the submitted version.

## Funding

This work was supported by the Chongqing Science and Health Joint Medical Research Project: 2018QNXM017 and 2022QNXM013.

## Conflict of Interest

KJ, CH, and JX were employed by Beijing Deepwise & League of PHD Technology Co. The remaining authors declare that the research was conducted in the absence of any commercial or financial relationships that could be construed as a potential conflict of interest.

## Publisher's Note

All claims expressed in this article are solely those of the authors and do not necessarily represent those of their affiliated organizations, or those of the publisher, the editors and the reviewers. Any product that may be evaluated in this article, or claim that may be made by its manufacturer, is not guaranteed or endorsed by the publisher.
